# Digital messaging to support control for type 2 diabetes (StAR2D): a multicentre randomised controlled trial

**DOI:** 10.1186/s12889-021-11874-7

**Published:** 2021-10-21

**Authors:** A. Farmer, K. Bobrow, N. Leon, N. Williams, E. Phiri, H. Namadingo, S. Cooper, J. Prince, A. Crampin, D. Besada, E. Daviaud, L-M Yu, J. N’goma, D. Springer, B. Pauly, L. Tarassenko, S. Norris, M. Nyirenda, N. Levitt

**Affiliations:** 1 Nuffield Department of Primary Care Health Sciences, University of Oxford, Oxford, UK; 2 Chronic Disease Initiative for Africa, University of Cape Town, Cape Town, South Africa; 3 Health Systems Research Unit, South-African Medical Research Council, Cape Town, South Africa; 4 Malawi Epidemiology and Intervention Research Unit, Lilongwe, Malawi; 5 Cochrane South Africa, South African Medical Research Council, Cape Town, South Africa; 6 Institute of Biomedical Engineering, Oxford, UK; 7 Kamuzu Central Hospital, Lilongwe, Malawi; 8 Boston Consulting Group, London, UK; 9 Department of Diabetes and Endocrinology, Chris Hani Baragwanath Academic Hospital, Johannesburg, South Africa; 10 Human Nutrition Unit, South African Medical Research Council, Johannesberg, South Africa

**Keywords:** Digital health, Adherence, Type 2 diabetes, Randomised trial, Sub-Saharan Africa, SMS text-messages

## Abstract

**Background:**

Failure to take medicines for diabetes as prescribed contributes to poor outcomes from the condition. Mobile phones are ubiquitous and short message service (SMS) texts have shown promise as a low-cost intervention. We tested the effectiveness of SMS-text messaging in improving outcomes in adults with type 2 diabetes.

**Methods:**

StAR2D was a 12-month two-arm randomised trial of SMS-text messaging and usual care in Cape Town, South Africa and Lilongwe, Malawi. Messages used behaviour change theory and were developed with patients and staff. The intervention group received four messages each week. The primary outcome was change in HbA1c. Secondary outcomes were the proportion of patients who collected > 80% medication and changes in systolic blood pressure, lipids, cardiovascular risk, and the proportion of the participants reaching treatment goals.

**Results:**

The trial took place between 1 October, 2016 and 1 October 2018, 1186 participants were randomised to intervention (593) and control (593) groups. 91% of participants completed follow-up. There was a reduction in HbA1c (DCCT) in both groups but not in mean change (95% CI) between groups (− 0.08% (− 0.31 to 0.16) (IFCC − 0.82 mmol/mol (− 3.44 to 1.79). There was a small but not significant increase in the proportions of participants likely to have collected 80% or more of medication (Relative risk 1.11 (0.84 to 1.47; *P* = 0.47). There was a significant difference between groups in change in systolic blood pressure from baseline of 3.46 mmHg (1.48 to 5.44, *P* = 0.001) in favour of the intervention group. The between group difference in change in 10-year risk of coronary heart disease was − 0.71% (− 1.46 to 0.04, *P* = 0.064). The proportion of participants meeting treatment goals in the intervention group was 36.0% and in the control group 26.8% (Relative risk 1.36 (1.13 to 1.63, *P* = 0.001). Participants reported many challenges to adherence despite finding messages acceptable and useful.

**Conclusions:**

Whilst SMS text messages do not lead to improved glycaemia in these low-resource settings there appeared to be an impact on blood pressure and achievement of treatment goals but the mechanisms for this are unclear. Text messages alone, may be unsuccessful unless accompanied by health system strengthening and other forms of self-management support for type 2 diabetes.

**Trial registration:**

Trial registration: ISRCTN, ISRCTN70768808. Registered 1 July 2015, http://www.isrctn.com/I ISRCTN70768808.

**Supplementary Information:**

The online version contains supplementary material available at 10.1186/s12889-021-11874-7.

## Background

The increasing burden of type 2 diabetes and its associated poor health outcomes disproportionately affects populations in low- and middle-income countries (LMIC) [1–3]. Effective management for people with diabetes requires a system of care to be in place that includes availability of various health care providers, access to treatments for blood glucose control as well as control of blood pressure and cholesterol levels, support for self-management, and strategies to detect and manage known complications from this progressive chronic condition [4].

The difficulties for patients in not collecting or taking medications have been extensively described and include a range of concerns and anxieties, lack of social support, limited health literacy, and negative interactions with the health care systems and lack of support for self-management [5, 6]. However, even with well- systems of care in place, failure to take medicine as prescribed or to follow other recommendations around diet and lifestyle, often referred to as nonadherence, can result in a failure to deliver the benefits of effective medical treatments into better outcomes for individual patients [6, 7].

Some but not all, interventions delivered by short message service (SMS) text messaging have been effective in increasing adherence to treatment and improving health outcomes for a range of chronic health conditions [8–10]. A systematic review suggests significant benefits of SMS text messaging for medication support in type 2 diabetes, but identified substantial heterogeneity in study length, study design with respect to the intervention and the comparator, and in content of the intervention. Studies included both high- and low-income settings, however there are few controlled studies from sub-Saharan Africa [11], and no studies comparing interventions across different health systems [12]. Controlled studies of longer duration, the use of structured message development, and carried out in sub-Saharan Africa are needed to understand and guide the use of digital health interventions.

Following formative work to develop messages and refine message delivery, we tested the effectiveness of sending SMS text messages in improving health outcomes and medication adherence in patients with type 2 diabetes compared to usual care in two low- and middle-income countries, Malawi and South Africa. We also carried out a process evaluation using qualitative research methods alongside the trial to investigate participant responses and acceptability, and we examined the cost of the intervention through a formal cost analysis.

## Methods

### Study design and participants

The SMS-Text Adherence Support for Type 2 Diabetes (StAR2D) trial was a 12-month, two parallel-arm, individually randomised controlled trial done in two urban sites serving patients living in middle- and low-income settings in Southern Africa: Cape Town, South Africa, and Lilongwe, Malawi. In Cape Town, recruitment was from a large primary care clinic serving two low-income communities to the north of the city. In Lilongwe, where many of the population live in low-income informal settlements, recruitment was from a hospital-based outpatient clinic. Despite the difference in gross domestic product between South Africa (6374 USD) and Malawi (371 USD), in both settings, there is an aspiration for patients to receive health care free of charge alongside supply of a limited range of essential medicines at no cost to patients. These medications include those to reduce blood glucose and blood pressure. In both sites group diabetes education sessions are provided whilst attending clinics.

### Randomisation and masking

We used a remote Web-based randomisation program, Sortition (Oxford), minimising for time since diagnosis (< 7 or ≥ 7 years), age (< 55 or ≥ 55 years), gender, and trial site. Adults with type 2 diabetes were allocated in a 1:1 ratio to receive automated text message support plus usual care or to usual care supplemented by active control. Allocations were directly uploaded into the trial database to avoid creation of locally held records. Randomisation was carried out remotely and independently of the clinic and local research staff. No arrangements for unblinding during the trial were made. We asked participants not to share the content of their text messages and did not recruit more than one participant from the same household. Clinic staff did not have access to information about allocated groups and did not have the facility to send text messages to individuals through the study system. Study procedures were carried out by trained research staff blinded to participant allocation group. Medication dispensing data were collected blind to participant allocation. Study outcomes were assessed by laboratory staff with no knowledge of treatment allocation or by research staff trained not to ask questions that would elicit group allocation.

### Intervention

The intervention was automated and consisted of motivational and educational text-messages sent to participants on different days, three to four times weekly over a period of 12 months. The intervention development process was intended to ensure the final brief (SMS) text-message intervention was theory- and evidence-informed, relevant, and acceptable to the target audiences, and appropriately aligned with the organisation of clinic care at the trial sites. Message content was intended to encourage people to take their medicine regularly as prescribed (70% of the messages), alongside other information intended to provide advice about healthy lifestyle and enhancing well-being (30% of the messages). Specific messages encouraged people to check the date of their next appointment and whether they had sufficient medication. Message content was developed from lived experience of diabetes, diabetes treatment services and expert opinion and formulated as SMS text-messages. The message development was guided by the Capability, Opportunity, Motivation-Behaviour model and described in detail a separate publication [13, 14]. Messages were translated into Xhosa, Afrikaans, Chichewa and English. Examples of messages used are given in supplementary appendix Table 1. Messages were randomly selected from a library, using rules that ensured individual messages were not repeated. Messages were personalised by using information given about smoking and use of alcohol to determine whether these messages were sent. Messages were tailored by having participants select the language in which to receive messages, and days on which they did not wish to receive messages. The messaging system allowed participants to change the delivery time or language of messages.

Participants allocated to the usual care group were sent a research related message thanking them for taking part in the study and providing trial related information every 6 weeks.

### Procedures

Both groups received a message welcoming them to the trial; instructions on how to change the phone number to receive messages and how to withdraw from the study, a security reminder that we would never ask for banking or personal information, a “happy birthday” message, and reminders to attend end of study visits. At both study sites, routine clinical care consisted of attendance to collect medication supplies at regular intervals. Routine review appointments were recommended biannually in Cape Town and annually in Lilongwe and included screening for complications associated with type 2 diabetes carried out according to the clinical guidelines in place at the clinic sites. Health material on type 2 diabetes was available at all sites, and included information about the importance of taking medicine regularly, alongside other health information.

Eligible patients attending clinics at both sites were those with type 2 diabetes; aged 18 years or greater; taking an oral glucose-lowering medication; able to communicate in one of the predominant official languages spoken in the Western Cape province in South Africa (English, Afrikaans, or isiXhosa) and in Malawi (English or the Chichewa language); with access to a mobile phone, where shared access is allowed with permission of the phone owner; ability to send and receive, or be helped to send and receive text messages; and current and planned future residence in participating clinic communities. Patients were not eligible for recruitment if they had been admitted to hospital for hyperglycaemia or hypoglycaemia within the previous 3 months; were pregnant or within 3 months postpartum by self-report or with plans to become pregnant in the next 12 months; had been diagnosed with a terminal medical condition; lived with another member of the household already recruited to the trial; or had participated in formative work.

Potential trial participants were approached sequentially by research staff whilst attending scheduled diabetes care sessions at the clinics. They received verbal and written information about the trial in their preferred language and had the opportunity to speak to trained study research staff and ask them questions. Initially, all participants provided verbal assent to screening procedures on agreeing to participate. If eligible, they then all provided written consent for enrolment in the trial. Copies of the consent forms were given to participants and randomisation was not carried out until they confirmed they had received a “welcome” text message.

Trial data collection was carried out using a low-cost mobile phone for data transmission. The phone ran a secure implementation of Sana Mobile (MIT). The Sana Mobile system used secure information exchange protocols to link to a secure server running Open Medical Records System (OpenMRS.org). The use of this system allowed checking of errors in data entry in real-time and enabled data to be immediately uploaded to the trial server.

Participants were invited by SMS text-message to attend the 12-month final trial assessment which was planned to coincide with their routine health care review. Participants who did not attend the 12-month follow-up clinic appointment were followed up using the mobile phone and other contact details. If the cause of nonattendance was death, then hospital records were obtained for review.

Data were collected by a team of research assistants trained and supervised to ensure consistency between and within study sites with standard operating procedures for clinical measurement. Data collection took place in clinics, community centres, and in participants homes. Access to end of study data was restricted to data management staff and trial statisticians.

The process evaluation will be reported in detail elsewhere. Briefly, we collected data at both sites using semi structured interviews and focus groups. We used purposive sampling to explore variation in response by variables, including age, gender, and language group of patient participants. We also developed interview guides to explore participant experience and views of receiving SMS text messages doing interviews at baseline (baseline interview guide in supplementary file 2), after enrolment and before a participant is randomised, and in interviews and focus groups with the same group of participants at the end of the trial (final interview guide in supplementary file 3). Trained qualitative researchers conducted the in-depth interviews and focus groups; data was captured with field notes and digital voice recording and was transcribed with anonymisation. Notes and transcripts were coded and themes developed. Standard approaches to ensuring the quality of the methodology were used, including dual review of interview summaries, use of a coding framework, and assessment of samples of dual-coded data.

### Deviations from protocol

Some participants taking insulin but not an oral glucose lowering medication were inadvertently recruited and randomised. These individuals were excluded from the analysis. The planned collection of data and notification of participants where medication was out of stock and tracking of participant attendance to collect medication were not implemented as planned. It emerged during the trial that procedures for dispensing and tracking medication identified in the formative work were no longer being used. We therefore adapted the original planned message to send as a prompt around the time a participant was calculated to require a medication refill, to check the status of their clinic appointment/medication refill. To streamline follow up we made the decision not to record self-reported diet and physical activity measures at follow up.

### Outcomes

We pre-specified change in HbA1c from baseline to 1 year as the primary outcome. Secondary outcomes were the proportion of patients collecting 80% or more of their agreed-upon, diabetes-related medication derived from routine clinic data [15]; change in systolic blood pressure; change in lipids; a combined measure of cardiovascular risk based on HbA1c, lipids, and systolic blood pressure [16]; and the proportion of the participants reaching treatment goals (HbA1c ≤8% and systolic blood pressure < 140 mmHg).

The EuroQol 5-Dimension 3-Level (EQ-5D-3L) instrument [17], and a locally adapted questionnaire, to establish satisfaction with treatment and delivery of treatment [18, 19], were used and available in all of the study languages. In addition, self-reported medication-taking was recorded [20]. Basic demographic data collected included age, gender, language preference, and work status. Anthropometric measures were collected, including measurement of height and weight using standard procedures. Full specification of secondary outcomes and methods of assessment have been previously published [21].

Outcomes of the process evaluation aimed to determine the factors (including individual, health system and broader contextual factors) influencing the impact of the intervention. Detailed economic findings will be reported in a separate paper.

### Sample size calculations

We estimated that a target sample of 1066 adults (including a loss to follow up of 20% and potential clustering between sites) would provide 90% power at 5% level of significance (2-sided) to show a reduction of 0.5% (5.5 mmol/mol) in HbA1c, assuming a standard deviation of 2.2% (24 mmol/mol).

### Statistical analysis

The study results are reported in accordance with the Consolidated Standards of Reporting Trials (CONSORT) 2010 statements [22]. A full detailed statistical analysis plan was prepared and finalised before participant follow-up was completed.

The results from the trial are presented as comparative summary statistics with 95% confidence intervals. All the tests were done at a 5%, two-sided significance level. Analysis of intervention effect was carried out on all participants with non-missing outcome data and participants were analysed in the groups to which they were allocated, regardless of what they actually received, but those randomised but recruited in error, who were not using an oral glucose lowering medication were excluded.

Comparison of change in HbA1c from baseline to 1 year between the intervention and usual care groups used a linear regression model, adjusting for baseline HbA1c and minimising variables. The treatment effect was presented as the adjusted difference between groups in the change in HbA1c from baseline, along with a 95% confidence interval and *P*-value derived from the corresponding t-statistic. Similar methods were used to analyse blood pressure data and other continuous outcomes.

We estimated the proportion of scheduled medication collection appointments successfully completed, in the following way, using routine registers maintained in the clinics. In Cape Town, data were obtained from two sources: (i) record sheets for centrally dispensed medication, delivered to clinics to be picked up by participants were used where marked to indicate collection; (ii) medical records were endorsed where prescriptions were locally dispensed with the participant in attendance. In Lilongwe, the data on participants attending were prospectively collected from clinic records. Records of duration of medication given were not available consistently, we therefore used a conservative estimate of medication being dispensed at three-monthly intervals. We then estimated, for each participant, the proportion of required medication likely to be available over 1 year from the attendance data. The proportions of participants who collected medication on at least 80% of scheduled occasions were compared using a log-binomial regression model adjusted for minimising variables. The intervention effect was presented as a relative risk with 95% confidence interval and *P*-value derived from the corresponding Z-statistic.

Missing data were reported with reasons given where available and the missing data pattern and mechanism were explored. We also did a sensitivity analysis using multiple imputation to examine the robustness of the results.

We did pre-specified subgroup analyses of the primary outcome and adherence outcome for the following subgroups: age (< 55 years or ≥ 55 years); site (Cape Town or Lilongwe); gender (male or female); number of years with type 2 diabetes (< 7 years or ≥ 7 years); presence of one or more comorbidity (none, one or more); diabetes control at baseline (HbA1c ≤53 mmol/mol or > 53 mmol/mol); and self-reported adherence rating score at baseline (25 or < 25). All statistical analyses were done using Stata version 15.1 (StataCorp LLC, College Station, USA).

### Laboratory measurement

HbA1c was measured using International Federation of Clinical Chemistry calibrated analysers linked to an international quality assurance scheme at both sites. Total and high-density lipoprotein (HDL) cholesterol was analysed using an enzymatic colorimetric method, again with an international quality assurance scheme.

### Data management

Electronic data capture was done with Sana mobile (MIT) using low-cost Android mobile phones and a real-time, mobile Internet connection to a server where the data was stored using OpenMRS [21].

### Approvals

The trial received ethical approval from the University of Oxford Tropical Research Ethics Committee (22-15), the University of Cape Town Human Research Ethics Committee (126/2015) and the Malawi National Health Services Research Committee (15/7/1425). All participants provided informed consent. The trial protocol has been previously published [21].

Data collection, management and analytical procedures were monitored by an independent data monitoring committee. Trial management was also reviewed by an independent trial steering committee.

## Results

Trial recruitment began in September 2016 and follow up of participants finished in October 2018. Of the 1930 potential participants screened, 744 were found to be ineligible.

**Fig. 1:**
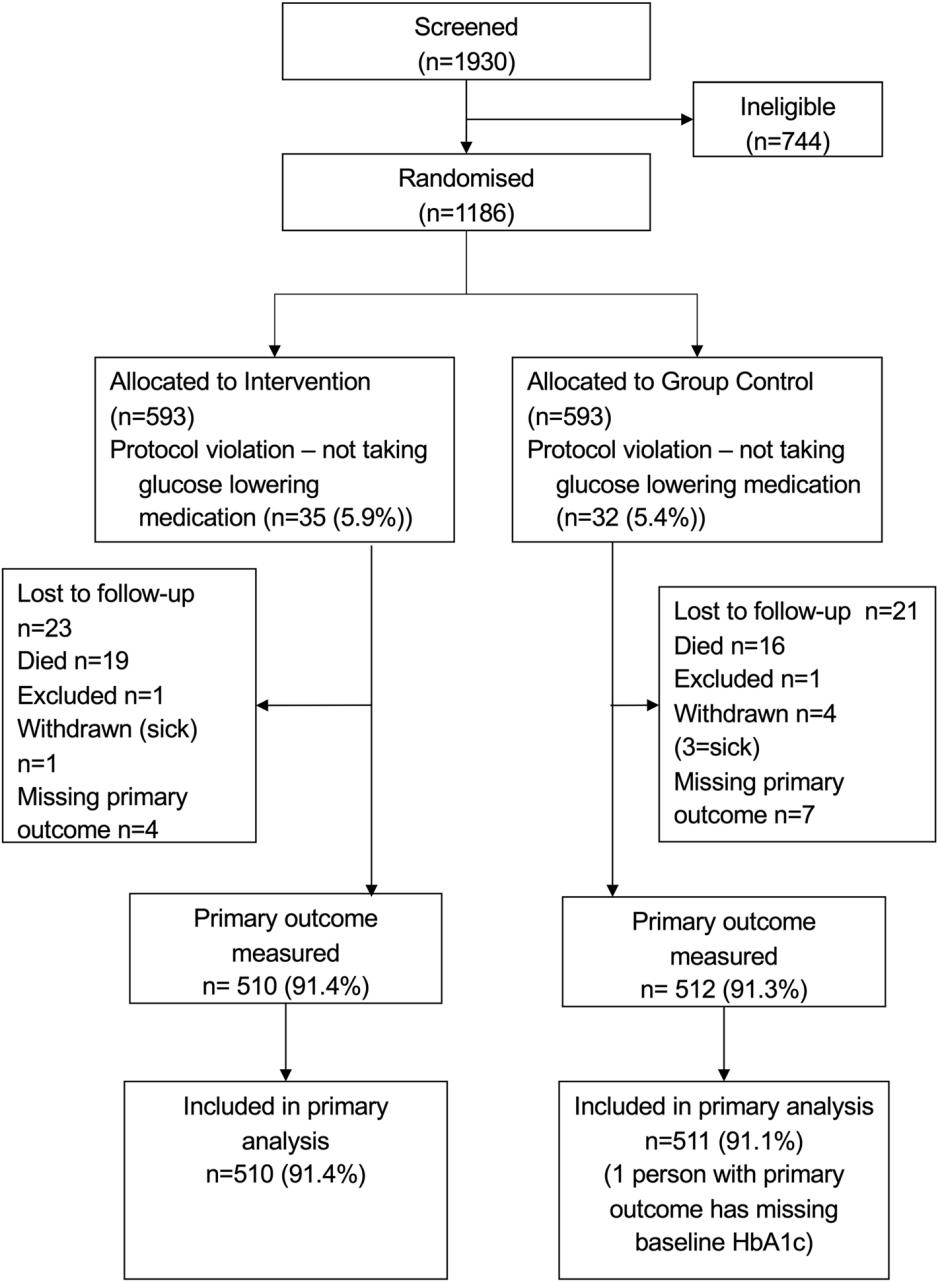
Heading: Trial Profile

In total, 1186 participants were randomly allocated to intervention (593) and control (593) (Fig. 1). There were 67 participants recruited who were subsequently identified as ineligible through using insulin alone without an oral glucose lowering drug and these were excluded. Baseline data were collected from all participants with the exception that 21 participants (for example, those in wheelchairs), could not have their weight measured with the measuring devices available.

Baseline characteristics were similar between allocated study groups (Table 1). Mean (SD) age was 57.1 (11.4) years, Body Mass Index 30.7 (7.0) kg/m2, years in school 9.0 (4.0), HbA1c 10.2 (3.5) %, systolic blood pressure 131.2 (20.4) mmHg, total cholesterol to HDL cholesterol ratio 4.4 (1.6). Of participants, 782 were women (69.9%) and 568 (50.8%) were based in Cape Town and 551 (49.2%) based in Lilongwe. The median (Q1, Q3) duration of diabetes was 5.0 (2.8, 10.0) years, and 10-year risk of coronary heart disease was 9.4 (4.7, 19.3) %. Mean baseline HbA1c differed between sites. The mean (SD) HbA1c for Cape Town was 9.1 (2.3) and for Lilongwe 11.3 (4.0)%.

**Table 1:** Baseline data

	Intervention *n* = 558	Control *n* = 561	Overall *n* = 1119
**Gender, n(%)**			
Female	390 (69.9)	392 (69.9)	782 (69.9)
Male	168 (30.1)	169 (30.1)	337 (30.1)
^a^**Age (years), n(%)**			
< 55 years	229 (41.0)	236 (42.1)	465 (41.6)
≥ 55 years	328 (58.8)	324 (57.8)	652 (58.3)
Missing	1 (0.2)	1 (0.2)	2 (0.2)
**Age (years)**			
Mean (SD)	56.8 (11.6)	57.4 (11.1)	57.1 (11.4)
Range	24.8 to 97.0	28.7 to 89.6	24.8 to 97.0
Missing	1	1	2
**Site, n(%)**			
Cape Town, SA	276 (49.5)	292 (52.1)	568 (50.8)
Lilongwe, Malawi	282 (50.5)	269 (48.0)	551 (49.2)
^a^**Duration of diabetes (years), n(%)**			
< 7 years	333 (59.7)	332 (59.2)	665 (59.4)
≥ 7 years	224 (40.1)	228 (40.6)	452 (40.4)
Missing	1 (0.2)	1 (0.2)	2 (0.2)
**Duration of diabetes (years)**			
Median	5.0	5.2	5
IQR	2.5 to 10	3 to 10.0	2.8 to 10
Missing	1	1	2
**Years in school/education**			
Mean (SD)	9.0 (3.9)	9.1 (4.2)	9,0 (4.0)
Missing	1	1	2
**Currently smoke, n(%)**			
Yes	68 (12.2)	72 (12.8)	140 (12.5)
No	489 (87.6)	488 (87.0)	977 (87.3)
Missing	1 (0.2)	1 (0.2)	2 (0.2)
**Currently use smokeless tobacco, n(%)**			
Yes	22 (3.9)	21 (3.7)	43 (3.8)
No	535 (95.9)	539 (96.1)	1074 (96.0)
Missing	1 (0.2)	1 (0.2)	2 (0.2)
**Tobacco Frequency, n(%)**			
Less than once a week	546 (97.9)	544 (97.0)	1090 (97.4)
Twice a week	0	2 (0.4)	2 (0.2)
3 to 5 times a week	0	2 (0.4)	2 (0.2)
Every or almost every day	7 (1.3)	9 (1.6)	16 (1.4)
More than once a day	4 (0.7)	3 (0.5)	7 (0.6)
Missing	1 (0.2)	1 (0.2)	2 (0.2)
**Live with a smoker, n(%)**			
Yes	157 (28.1)	154 (27.5)	311 (27.8)
No	399 (71.5)	402 (71.7)	801 (71.2)
Unsure	1 (0.2)	4 (0.7)	5 (0.5)
Missing	1 (0.2)	1 (0.2)	2 (0.2)
**BP (mmHg)**			
Systolic, Mean (SD)	130.6 (20.4)	131.7 (20.4)	131.2 (20.4)
Diastolic, Mean (SD)	78.3 (10.9)	79.0 (11.4)	78.7 (11.1)
Missing	1	2	3
**BMI (kg/m**^**2**^**)**			
Mean (SD)	30.6 (6.5)	30.8 (7.4)	30.7 (7.0)
Missing	8	12	20
**HbA1c (%)**			
Mean (SD)	10.1 (3.4)	10.2 (3.6)	10.2 (3.5)
Missing	1	3	4
**HbA1c (mmol/mol)**			
Mean (SD)	87.1 (36.8)	88.4 (38.9)	87.7 (37.8)
Missing	1	3	4
**HDL Cholesterol (mmol/l)**			
Mean (SD)	1.2 (0.4)	1.2 (0.3)	1.2 (0.3)
Missing	1	2	3
**Total Cholesterol (mmol/l)**			
Mean (SD)	4.9 (1.3)	4.9 (1.3)	4.9 (1.3)
Missing	4	4	8
**Ratio of HDL to Total Cholesterol**			
Mean (SD)	0.25 (0.09)	0.25 (0.08)	0.25 (0.08)
Missing	4	4	8
**SMS Language, n(%)**			
Chichewa	252 (45.2)	246 (43.9)	498 (44.5)
Isixhosa	109 (19.5)	108 (19.3)	217 (19.4)
English	112 (20.1)	120 (21.4)	232 (20.7)
Afrikaans	84 (15.1)	86 (15.3)	170 (15.2)
Missing	1 (0.18)	1 (0.18)	2 (0.18)
**Current health, n(%)**			
Poor	44 (7.9)	52 (9.3)	96 (8.6)
Fair	163 (29.2)	172 (30.7)	335 (29.9)
Good	303 (54.3)	291 (51.9)	594 (53.1)
Excellent	47 (8.4)	45 (8.0)	92 (8.2)
Missing	1 (0.18)	1 (0.18)	2 (0.18)
**Medication use, n(%)**			
Insulin	91 (16.3)	102 (18.2)	193 (17.3)
Statin	216 (38.7)	243 (43.3)	459 (41.0)
Metformin	527 (94.4)	528 (94.1)	1055 (94.3)
Sulfonylurea	396 (71.0)	378 (67.4)	774 (69.2)
Pioglitazone	0	0	0
BP lowering medication	394 (70.6)	416 (74.2)	810 (72.4)
Missing	1 (0.18)	1 (0.18)	2 (0.18)
**MARS-5**^b^			
Mean (SD)	22.7 (2.6)	22.7 (2.8)	22.7 (2.7)
Missing	1	1	2
**EQ5D-3 L Index Value**			
Mean (SD)	0.78 (0.26)	0.78 (0.27)	0.78 (0.26)
Missing	1	1	2
**Satisfaction with health care**			
Mean (SD)	4.2 (0.8)	4.2 (0.8)	4.2 (0.8)
Missing	1	1	2
**Self-reported eating**			
Mean (SD)	11.8 (4.9)	11.4 (5.0)	11.6 (4.9)
Missing	1	1	2
**Self-reported physical activity**			
Mean (SD)	7.4 (6.0)	6.7 (5.8)	7.0 (5.9)
Missing	1	1	2
**10-year coronary heart disease risk (%)**			
Median	9.1	9.6	9.4
IQR	4.4 to 19.4	4.8 to 19.3	4.7 to 19.3
Missing	4	6	10
**Number of glucose lowering medications prescribed**			
Mean (SD)	1.8 (0.5)	1.8 (0.5)	1.8 (0.5)
Median (IQR)	2 (2 to 2)	2 (1 to 2)	2 (2 to 2)
Missing	1	2	3
**Number of blood pressure lowering medications prescribed**			
Mean (SD)	1.5 (1.2)	1.5 (1.2)	1.5 (1.2)
Median (IQR)	2 (0 to 2)	2 (0 to 2)	2 (0 to 2)
Missing	1	2	3
**Number of cholesterol lowering medications prescribed**			
Mean (SD)	0.4 (0.5)	0.4 (0.5)	0.4 (0.5)
Median (IQR)	0 (0 to 1)	0 (0 to 1)	0 (0 to 1)
Missing	1	2	3
**Comorbidities, n(%)**			
**Heart attack**	36 (6.5)	28 (5.0)	64 (5.7)
**Heart failure**	28 (5.0)	20 (3.4)	48 (4.3)
**TIA/mini stroke**	45 (8.1)	41 (7.3)	86 (7.7)
**Angina/chest pain**	50 (9.0)	41 (7.3)	91 (8.1)
**Peripheral vascular disease**	79 (14.2)	65 (11.6)	144 (12.9)
**Chronic kidney disease**	21 (3.8)	18 (3.2)	39 (3.5)
**TB**	47 (8.4)	58 (10.3)	105 (9.4)
**Mental Illness**	25 (4.5)	29 (5.2)	54 (4.8)

^a^Variables used in minimisation

^b^Medication adherence report scale

The primary outcome was measured in 510 (91.4%) of those in the intervention group and 512 (91.3%) of those in the control group. Follow up was similar across both sites.

Exposure to the intervention was high, with a high overall rate of delivery of SMS text messages. Out of a total of 114,207 SMS messages, only 7644 (6.7%) were not sent from the server or not delivered.

**Fig. 2:**
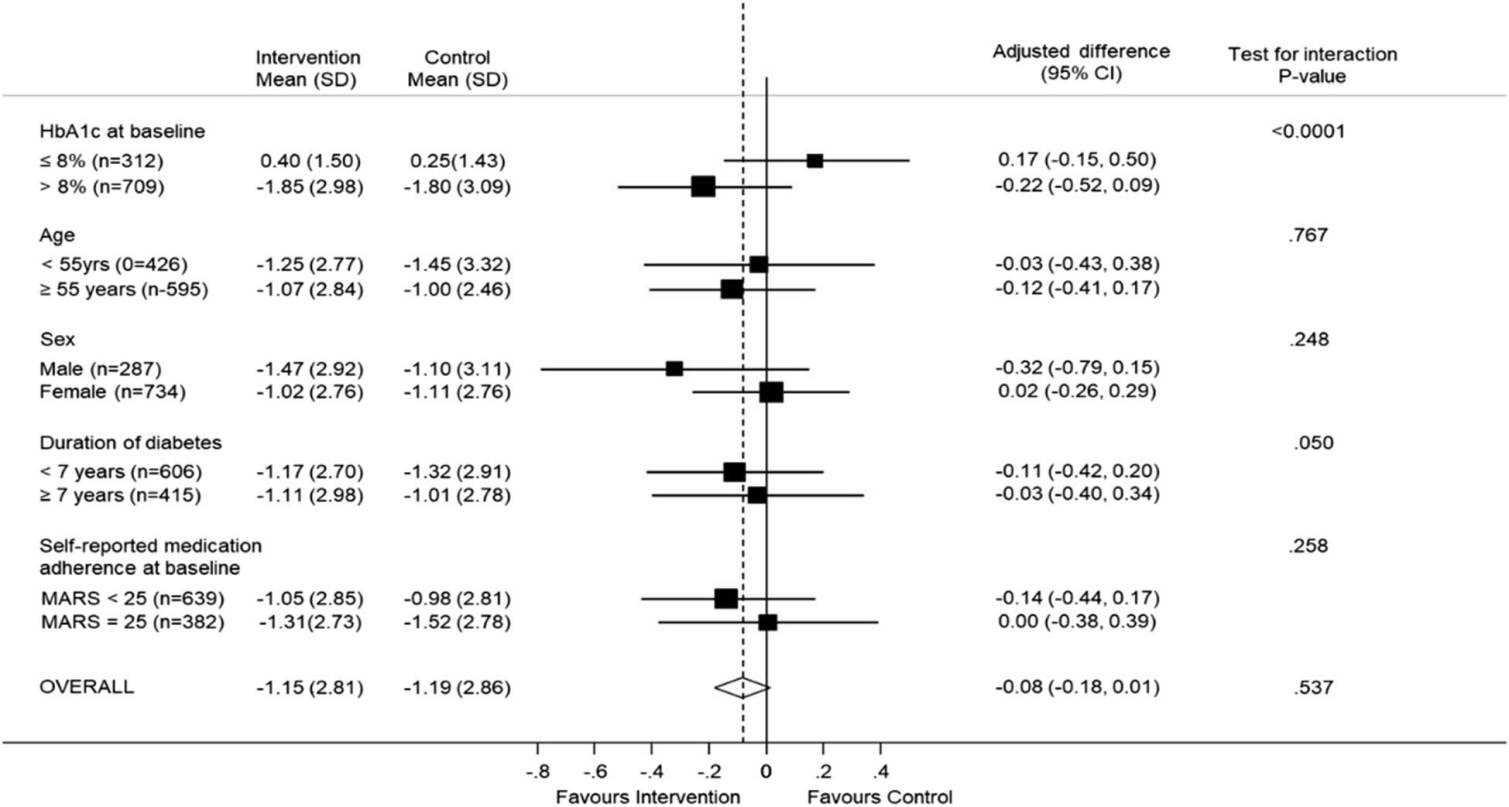
Treatment effect on primary outcome (HbA1c mmol/mol) and treatment effect on primary outcome by subgroup

The HbA1c fell in both the intervention and usual care groups, and there was no difference in the change in HbA1c between intervention and usual care groups. The change (reduction) in HbA1c (SD) DCCT [IFCC] from baseline to 1 year was − 1.15 (2.81) % [− 12.53 (30.72) mmol/mol] in the intervention group and − 1.19 (2.86) [− 13.02 (31.27) mmol/mol] in the control group (Fig. 2). The overall adjusted difference in HbA1c (95% CI) between groups was − 0.08% (− 0.31 to 0.16) [− 0.82 (− 3.44 to 1.79) *P* = 0.537] in favour of the intervention group. We observed no differences in medication use over time between groups (supplementary tables).

There was a reduction in systolic blood pressure of 0.17 mmHg in the intervention group and a rise of 3.11 mmHg in the control group, with a between group difference of − 3.46 mmHg (− 5.44 to − 1.48, *P* = 0.001) in favour of the intervention group (Table 2). We observed no differences in medication use over time between groups (supplementary tables). The corresponding difference in change in 10-year risk of coronary heart disease was − 0.71% (− 1.46 to 0.04, *P* = 0.064). The proportion of participants meeting treatment goals at one-year in the intervention group was 36.0% and in the control group 26.8% (Relative risk 1.36 (1.13 to 1.63, *P* = 0.001). There were no other clinically important between group differences in the clinical, self-report measures of medication use and satisfaction.

**Table 2:** Treatment effect on secondary outcomes

Outcomes				
	Change from baseline to 12 months			
	Intervention	Control	Mean difference	P
Systolic blood pressure	− 0.17 (18.9)	3.11 (19.24)	−3.46 (− 5.44 to − 1.48)	0.001
Total/HDL cholesterol	−0.03 (1.3)	0.01 (1.1)	−0.03 (− 0.17 to 0.10)	0.633
Body mass index (kg/m2)	0.03 (2.14)	−0.07 (3.76)	−0.08 (− 0.45 to 0.29)	0.669
Modelled cardiovascular risk	−1.24 (8.22)	−0.38 (7.94)	− 0.71 (− 1.46 to 0.041)	0.064
Health status EQ-5D	0.009 (0.160)	−0.004 (0.157)	− 0.011 (− 0.007 to 0.030)	0.202
Medication taking (MARS)	0.6 (2.81)	0.37 (3.18)	−0.26 (− 0.03 to 0.54)	0.075
Satisfaction with health care	0.27 (0.74)	0.21 (0.74)	−0.030 (− 0.038 to 0.099)	0.385
	Intervention	Control	Relative Risk (95% CI)	P
Reaching treatment goals	182 (36.0)	136 (26.8)	1.36 (1.13 to 1.63)	0.001
≥80% visits for medication collection	87 (15.7)	79 (14.2)	1.11 (0.84 to 1.47)	0.469

Data on self-reported dietary intake and physical activity was not recorded at follow up

There was no evidence of impact on the secondary outcome of medication adherence. Our pre-specified cut-off for adherence of attending at least 80% of scheduled medication collection appointments was met by only 15.7% of the intervention group and 14.2% of the control group (Relative risk 1.11 (0.84 to 1.47; *P* = 0.47). In addition, we carried out an exploratory analysis of adherence using continuous data. This did not change the conclusion with a mean (SD) percentage of the proportion of diabetes-related medication collected over 1 year of 56.7 (23.0)% for the intervention group and 54.2 (23.0)% for usual care (*P* = 0.061).

The pre-specified subgroup analyses (Table 3 and Fig. 2) for HbA1c outcomes suggests an interaction between change in HbA1c for those with a HbA1c > 53 mmol/mol and ≤ 53 mmol/mol. There is a difference between intervention and control of HbA1c (DCCT) in favour of intervention of − 1.85 mmol/mol for HbA1c > 53 mmol/mol and a difference of 0.4 mmol/mol in favour of control for ≤53 mmol/mol (*P*-value interaction < 0.0001). There was a marginal interaction for change in HbA1c by duration of diabetes (*P* = 0.05). There was no evidence of interactions for diabetes control, by site, age, gender, or presence of comorbidities. There were 19 (3.4%) deaths in the intervention group and 16 (2.9%) deaths in the control group. There were no adverse events related to the trial intervention.

**Table 3:** Treatment effect on primary outcome and treatment effect on primary outcome by subgroup

Change in HbA1c (mmol/mol) from baseline to 12 months				
	Intervention^a^	Control^a^	Mean difference^b^	P
HbA1c (mmol/mol)	−12.5 (30.72)	−13.0 (31.27)	−0.82 (−3.44 to 1.79)	0.537
*HbA1c at baseline*				
≤ 64 mmol/mol	4.4 (16.39)	2.7 (15.68)	1.89 (−1.68 to 5.46)	< 0.0001
> 64 mmol/mol	−20.3 (32.59)	− 19.7 (33.76)	−2.36 (−5.70 to 0.97)	
*Age*				
< 55 years	−13.7 (30.29)	−15.8 (36.32)	−0.28 (−4.70 to 4.14)	0.767
≥ 55 years	−11.7 (31.05)	− 11.0 (26.90)	−1.30 (− 4.50 to 1.90)	
*Gender*				
Male	−16.1 (31.88)	−15.3 (33.96)	−3.47 (−8.61 to 1.67)	0.248
Female	− 11.1 (30.18)	−12.1 (30.17)	0.17 (−2.88 to 3.22)	
*Duration of diabetes*				
< 7 years	−12.8 (29.48)	−14.4 (31.86)	− 1.20 (−4.62 to 2.22)	0.050
≥ 7 years	− 12.2 (32.53)	−11.0 (30.37)	−0.37 (− 4.42 to 3.68)	
*Self-reported medication adherence at baseline*				
MARS < 25	−11.5 (31.18)	− 10.7 (30.74)	− 1.51 (− 4.86 to 1.84)	0.258
MARS = 25	−14.4 (29.85)	− 16.6 (31.83)	0.05 (− 4.18 to 4.29)	

^a^Mean (SD)

^b^Mean (95% CI)

### Process evaluation

Details of our qualitative findings will be reported elsewhere. In summary, participants in the intervention arm of the trial found brief text messaging for diabetes adherence acceptable and useful for addressing informational and support needs. There were some examples of positive behaviour change reported due to the text reminders and advice on healthy lifestyle. There was a strong relational effect with messages and trial participation being experienced as a source of support, caring and motivation. However, participants’ ability to act on the messages was limited, against a background of navigating a complex set of challenges in coping with their diabetes and other life challenges. Impact and participant responses were similar across sites, despite socio-economic and health service differences. Common themes were participants’ frustration over how unstable their health was, their struggle to control their blood glucose levels, and their need for more responsive health care.

### Costs

The annualised economic cost of the study intervention amounted to $7883 in the Malawi clinic and $6336 in the South African clinic. Fixed costs (formative research, set-up, and programming) represented respectively 72 and 83% of these costs. This difference is largely explained by the fact that the cost of SMS text-messages in Malawi is twice the cost of SMS in South-Africa. If the intervention was scaled-up and rolled out to 90% of adults with diabetes on treatment in each country, the shares of fixed costs decrease very significantly to 1.33% in Malawi and 0.04% in South-Africa, the largest cost becoming that of the SMS text-messages. The annualised cost of the intervention per diabetes patient year would amount to $6.47 in Malawi and $3.16 in South-Africa. The cost per patient year of routine treatment is $58.58 and $189.96 respectively. Including the intervention as part of routine treatment would thus increase the cost per patient year by 11.05% in Malawi and 1.66% in South Africa. Costs to patients affect adherence. For patients using private and public transport, (95% in Lilongwe and 42% in Cape Town) transport cost per visit was the equivalent of 5 loaves of bread in Lilongwe and 2.5 in Cape Town. Time in clinic was significant, mainly due to waiting time. It was an average of 2.5 h in Lilongwe and just under 5 h in Cape Town.

## Discussion

The StAR2D trial does not provide convincing evidence that well-designed text messages lead to changes in glycaemic control across the range of baseline HbA1c for people with type 2 diabetes and across two low resource settings in urban sub Saharan Africa, but there is evidence for a significant and clinically important impact on blood pressure and attainment of treatment goals in this population, although the reasons for a difference in impact on glycaemia and blood pressure are unclear. Findings were similar between two contrasting settings. The process evaluation suggested that the SMS messages were seen as relevant and useful, providing examples of behaviour change, although messages did not appear to address the complex reasons underlying ongoing partial adherence behaviours or the clinical need for escalating treatment. Set-up costs per patient become minimal when the intervention is scaled up. Then the main cost driver is that of cost per SMS message in the country.

We compared our study to those included in at least one of three recent meta-analyses of SMS texts to support people with diabetes [23–25]. We found most of the studies included were small (100 participants or less) of short duration (3 to 6 months), focused on people with poorly controlled HbA1c levels (defined as a HbA1c > 7%) and compared intervention to usual care, although usual care varied across studies. There was variation in the content and dosage of the intervention but no obvious dose-response pattern. In most of the studies, both intervention and control arms showed a fall in the level of HbA1c including those studies where the between group difference was not significant which is consistent with our findings. It is possible that willingness to take part in a trial may also be associated with changes in motivation relating to medication use and other lifestyle behaviours. Larger studies and those carried out over a longer period did not identify statistically significant differences in measures of HbA1c between intervention and control groups [23–25], with one widely reported exception [26]. This nine-month New Zealand study differed from ours, in that the participants were people with type 1 and type 2 diabetes, who were using oral glucose lowering medication and insulin and the intervention included graphical feedback of blood glucose readings [26]. The closed-loop communication between participants and providers in the New Zealand study may have enhanced the impact of the intervention. We involved clinicians in the design of the messaging system, but not, in day to day interaction with patients as this would not have been feasible either in our local context or to scale more widely. We observed that this may have reduced our agility in responding to local context.

The trial setting is representative of outpatient clinical care available for most people with type 2 diabetes in LMIC. The level of HbA1c in the population was high in both sites and despite the long duration of type 2 diabetes, many people were still on oral monotherapy and had not been prescribed additional treatment. The numbers of people who died during the 1 year of the trial emphasises the extent of morbidity and mortality in this population.

Our results showed mixed effects on outcomes that are important for people with type 2 diabetes. For example, we observed a similar, though larger effect of the intervention on lowering blood pressure than that reported in a previous study using SMS text-messaging to support the regular use of medication [27], but there was no similar impact on HbA1c. Some of the messages focussed on cardiovascular risk factors and cardiovascular disease prevention. In pre-specified sub-group analyses, we found evidence of interaction by the level of HbA1c at the start of the trial and by the duration of diabetes (self-reported time since diagnosis). The results suggest that it is possible that people with higher starting HbA1c and shorter duration of disease may have benefitted more from the intervention than those with lower baseline HbA1c or longer duration of disease. However, the effect is small and the mean difference in HbA1c is not non clinically relevant. These results show the limited scope for an effect from SMS text-messages on glycaemic control in the absence of treatment intensification or increased levels of support.

A cost-effectiveness analysis had originally been planned. However, findings showed no difference between intervention and control groups for the main outcome of the study and no difference in EQ-5D measures, so this analysis has not been done.

StAR2D tested at scale, the use of brief messages delivered by SMS text-message for improving outcomes for people with type 2 diabetes. HbA1c and blood pressure outcomes were collected blind to randomly allocated group and with minimal loss to follow up. The design of the study excluded interim HbA1c measures that may have identified an early, but transient change in HbA1c level (potentially at the expense of modifying the impact of the trial interventions) in contrast to other shorter-term studies. We did not, for similar reasons, have additional measures in place to self-report whether messages were read, but our formative work did not indicate this would be a problem, we tracked whether participants were receiving messages on their mobile phones, and they reported receiving and reading them in interviews after the trial had finished. We did not measure health literacy as a dependent variable and did not measure whether people had the messages read to them, but most participants interviewed in our qualitative study had basic knowledge, although lacked personal information about what might be needed to stabilise their blood sugar.

This trial provided a good test of the pragmatic impact of messaging without additional support beyond usual clinic care. As a test of efficacy, this study was limited by wide inclusion criteria including participants with comorbid conditions and those with a varied degree of glycaemic control. The study builds on previous work [27], with an increased frequency of sending messages and a message library utilising a wide range of behaviour-change techniques and an increased number of content domains [28].

## Conclusions

In this large, robust, randomised trial in two sub-Saharan African cities, we did not find convincing evidence that well-designed text messages lead to changes in glycaemic control for people with type 2 diabetes receiving usual primary care, although there was evidence for a clinically important impact on blood pressure and attainment of treatment goals. Text messages alone [11], may be unsuccessful in achieving glycaemic control unless accompanied by multi-modal health system strengthening and other forms of support for self-management. Further work is needed on what health service and self-management components are needed in combination with targeted digital communication using a wider range of approaches, to support diabetes adherence behaviour.

## Supplementary Information

**Additional file 1.** Collaborating Centres and supplementary data table.

**Additional file 2.** Baseline process evaluation interview guide.

**Additional file 3.** Final process evaluation interview guide.

## Data Availability

The datasets used and analysed are available from the corresponding author on reasonable request.

## Abbreviations

CONSORT: Consolidated Standards of Reporting Trials
EQ-5D-3L: EuroQol 5-Dimension 3-Level
HbA1c: Hemoglobin A1c
HDL: High-density lipoprotein
ICH GCP: International Conference on Harmonisation of Technical Requirements for Registration of Pharmaceuticals for Human Use-Good Clinical Practice
ICMJE: International Committee of Medical Journal Editors
ITT: Intention-to-treat
NHSRC: National Health Services Research Committee
OpenMRS: Open Medical Records System
OXTREC: University of Oxford Tropical Research Ethics Committee
SMS: Short message service
StAR2D: SMS-Text Adherence Support for Type 2 Diabetes
StAR-BP: SMS-Text Adherence Support-Blood Pressure
UCT HREC: University of Cape Town Research Ethics Committee
