# Correction to: Digital messaging to support control for type 2 diabetes (StAR2D): a multicentre randomised controlled trial

**DOI:** 10.1186/s12889-022-13085-0

**Published:** 2022-04-13

**Authors:** A. Farmer, K. Bobrow, N. Leon, N. Williams, E. Phiri, H. Namadingo, S. Cooper, J. Prince, A. Crampin, D. Besada, E. Daviaud, L-M Yu, J. N’goma, D. Springer, B. Pauly, L. Tarassenko, S. Norris, M. Nyirenda, N. Levitt

**Affiliations:** 1 Nuffield Department of Primary Care Health Sciences, University of Oxford, Oxford, UK; 2 Chronic Disease Initiative for Africa, University of Cape Town, Cape Town, South Africa; 3 Health Systems Research Unit, South-African Medical Research Council, Cape Town, South Africa; 4 Malawi Epidemiology and Intervention Research Unit, Lilongwe, Malawi; 5 Cochrane South Africa, South African Medical Research Council, Cape Town, South Africa; 6 Institute of Biomedical Engineering, Oxford, UK; 7 Kamuzu Central Hospital, Lilongwe, Malawi; 8 Boston Consulting Group, London, UK; 9 Department of Diabetes and Endocrinology, Chris Hani Baragwanath Academic Hospital, Johannesburg, South Africa; 10 Human Nutrition Unit, South African Medical Research Council, Johannesberg, South Africa

**Correction to: BMC Public Health 21, 1907 (2021)**

**https://doi.org/10.1186/s12889-021-11874-7**

In the original publication there was an incomplete funding acknowledgement. In this correction article the correct and incorrect funding acknowledgement are published. The original article has been updated.

**Incorrect funding**

- The trial was funded by the UK Medical Research Council (MR/M016498/1)) with the South African Medical Research Council under the Global Alliance for Chronic Disease Diabetes Programme. The trial sponsor was the Joint Research Office, University of Oxford, UK. The funder had no role in the design, execution, analyses, interpretation of the data, or decision to submit results.

**Correct funding**

- The trial was funded by the UK Medical Research Council (MR/M016498/1)) with the South African Medical Research Council under the Global Alliance for Chronic Disease Diabetes Programme. The trial sponsor was the Joint Research Office, University of Oxford, UK. The funder had no role in the design, execution, analyses, interpretation of the data, or decision to submit results.
- AF & LT were supported by the National Institute for Health Research (NIHR) Oxford Biomedical Research Centre (BRC). The views expressed are those of the author(s) and not necessarily those of the NHS, the NIHR or the Department of Health.

