# Supplementary material for: Digital messaging to support control for type 2 diabetes (StAR2D): a multicentre randomised controlled trial
Source: BMC Public Health. 2021 Oct 21;21:1907. doi: 10.1186/s12889-021-11874-7 (PMC8529732; doi:10.1186/s12889-021-11874-7)
Supplement: Supplementary file 1 — Additional file 1. Collaborating Centres and supplementary data table. [file 12889_2021_11874_MOESM1_ESM.docx]

Supplementary Appendix

This appendix has been provided by the authors to give readers additional information about their work.

Supplement to:

Digital Messaging to support control for type 2 diabetes (StAR2D): a multicentre randomized controlled trial.

Publication citation to be inserted

**Supplementary Appendix**

**Contents**

Writing Group.............................................................................................................................. 1

Local Investigators....................................................................................................................... 1

Steering Committee....................................................................................................................... 1

Data Monitoring Committee ........................................................................................................... 1

Senior Management Team ............................................................................................................ 1

Coordinating Office (University of Cape Town):............................................................................ 2

Formative and process evaluation ................................................................................................................... 2

Collaborating Centres ................................................................................................................... 2

Supplementary Table: Treatment effect on HbA1c by subgroup....................................................................... 3

.....

**Members of Star2D Trial Collaborative Group**

**Writing Group**

A Farmer*, K, Bobrow*, N Leon (NaL), N Williams, E Phiri, H Namadingo, S Cooper, J Prince, L M Crampin, D Besada, E Daviaud, L-M Yu, J N'goma, D Springer, B Pauly, Tarassenko, S Norris, M Nyirenda, N Levitt (NL).

* Equal first author.

Institutions: *Nuffield Department of* *Primary Care Health Sciences, University of Oxford* AF, L-MY, NW; *Institute of Biomedical Engineering, Oxford* LT, JP; *Health Systems Research Unit, South-African Medical Research Council* NaL, DB, ED; *Cochrane South Africa, South African Medical Research Council:* SC*; Chronic Disease Initiative for Africa, University of Cape Town* KB, NL; *Human Nutrition Unit, South African Medical Research Council* SN; *Department of Diabetes and Endocrinology, Chris Hani Baragwanath Academic Hospital, Johannesburg* BP; *Kamuzu Central Hospital, Lilongwe* JN; Boston Consulting Group, London DS; and the *Malawi Epidemiology and Intervention Research Unit, Lilongwe, Malawi* MC, MN, HN, EP.

**Local Investigators**

N Levitt, J N'Goma

**Steering Committee**

*Chair:* M Thorogood; *Chief investigator:* A Farmer; *Statisticians:* L-M Yu, N. Williams; *Administrative coordinators:* S Robinson V Madikizela; *Other members: N* Levitt, P Dorairaj, M Tomlinson, K Bobrow. A Harris representing MRC UK.

**Data Monitoring Committee**

*Chair:* V Cornelius; *Members:* M Bachman, R Mash.

**Senior Management Team**

*Lead:* A Farmer; *Members*: N Levitt, K Bobrow, M Crampin, N Leon.

**Coordinating Office (Chronic Diseases Initiative for Africa, University of Cape Town)**

*Administration and support:* V Madikizela, C Delport, L Fisher (coordinators).

***Statistics and computing support***

University of Oxford: J Prince, D Springer, N Nayan.

**Coordinating Office (Malawi Epidemiology and Intervention Research Unit)**

*Coordinator*: E Piri

**Formative and Process evaluation**

*Lead***:** N Leon**;** *Researchers:* H Namadingo, S Cooper, Namhla Sicwebu

**Collaborators**

*Vanguard Clinic, Bonteheuwel, Cape Town, South Africa*: M Namane*: Kamuzu Central Hospital, Lilongwe, Malawi:* J N'Goma

**Supplementary Table 1: Examples of SMS Messages sent to participants in the StAR2D trial participants allocated to the trial intervention arm.**

Taking your diabetes pills as directed means you are in control. Keep up the commitment to taking your meds!

Forgetting to take your medicine? Think about what is getting in the way & try to improve.

Keeping your meds in a place where you can see them (e.g. on the kitchen table) can help you remember to take them regularly.

It’s easy to forget your meds at home when travelling. Pls remember to take your meds along on your trip

Pls try talk to us if you think your meds are making you feel unwell.

Attending clinic appointments will help you stay healthy. Pls try to plan ahead for your arrangements (e.g., travel, work, child care).

Well-controlled blood sugar (by taking your meds) helps prevent heart disease.

There’s always more you can learn about diabetes. Keep educating yourself to take care of your health.

Having the support of family & friends can help you manage your diabetes. Ask people you trust to help you.

Moderate exercise may help you stay healthy. Try to make exercise part of your daily routine, such as walking.

**Supplementary Table 2: Treatment effect on primary outcome (HbA1c %) and treatment effect on primary outcome by subgroup (presented using DCCT units)**

**Outcomes**

Change in HbA1c (DCCT) from baseline to twelve months

Intervention* Control* Mean difference† P

HbA1c (DCCT %) -1.15 (2.81) -1.19 (2.86) -0.08 (-0.31 to 0.16) 0.537

*HbA1c at baseline*

≤8% 0.40 (1.50) 0.25 (1.43) 0.17 (-0.15 to 0.50) <0.0001

>8% -1.85 (2.98) -1.80 (3.09) -0.22 (-0.52 to 0.09)

*Age*

<55 years -1.25 (2.77) -1.45 (3.32) -0.025 (-0.43 to 0.38) 0.767

≥55 years -1.07 (2.84) -1.00 (2.46) -0.12 (-0.41 to 0.17)

*Gender*

Male, -1.47 (2.92) -1.40 (3.11) -0.32 (-0.79 to 0.15) 0.248

Female -1.02 (2.76) -1.11 (2.76) 0.02 (-0.26 to 0.29)

*Duration of diabetes*

< 7 years -1.17 (2.70) -1.32 (2.91) -0.11 (-0.42 to 0.20) 0.050

≥ 7 years -1.11 (2.98) -1.01 (2.78) -0.03 (-0.40 to 0.34)

*Self-reported medication adherence at baseline*

MARS < 25 -1.05 (2.85) -0.98 (2.81) -0.14 (-0.44 to 0.17) 0.258

MARS = 25 -1.31 (2.73) -1.52 (2.91) 0.005 (-0.38 to 0.39)

* Mean (SD)

† Mean (95% CI)

**Supplementary Table 3: Medication, difference between number taken at 12 months and number taken at baseline n (%)**

|  | **Intervention**  **n=558** | ***Control***  **n=561** |
| --- | --- | --- |
| **Oral glucose lowering** |  |  |
| 2  1  0  -1  -2  -3  Missing | 0  45 (8.1)  432 (77.4)  42 (7.5)  38 (6.8)  0  1 (0.2) | 1 (0.2)  41 (7.3)  426 (75.9)  52 (9.3)  38 (6.8)  1 (0.2)  2 (0.4) |
| **Insulin** |  |  |
| 2  1  0  -1  Missing | 4 (0.7)  16 (2.9)  522 (93.6)  15 (2.7)  1 (0.2) | 3 (0.5)  18 (3.2)  522 (93.1)  16 (2.9)  2 (0.4) |
| **Any glucose lowering** |  |  |
| 2  1  0  -1  -2  -3  -4  Missing | 1 (0.2)  57 (10.2)  417 (74.7)  44 (7.9)  35 (6.3)  3 (0.5)  0  1 (0.2) | 2 (0.4)  50 (8.9)  422 (75.2)  41 (7.3)  40 (7.1)  3 (0.5)  1 (0.2)  2 (0.4) |
| **Cholesterol lowering** |  |  |
| 1  0  -1  -2  Missing | 21 (3.8)  502 (90.0)  34 (6.1)  0  1 (0.2) | 19 (3.4)  491 (87.5)  48 (8.6)  1 (0.2)  2 (0.4) |
| **Blood pressure lowering** |  |  |
| 5  4  3  2  1  0  -1  -2  -3  -4  Missing | 0  0  2 (0.4)  2 (0.4)  52 (9.3)  268 (48.0)  170 (30.5)  52 (9.3)  11 (2.0)  0  1 (0.2) | 1 (0.2)  0  3 (0.5)  7 (1.3)  42 (7.5)  274 (48.8)  166 (29.6)  48 (8.6)  16 (2.9)  2 (0.4)  2 (0.4) |

The difference between baseline and 12 months in the number of drugs being taken in each class has been calculated (baseline-12 months). A positive difference indicates a reduced number of drugs being taken (by one or more drugs) and a negative difference indicates an increased number of drugs being taken.
